# MDR: an integrative DNA N6-methyladenine and N4-methylcytosine modification database for Rosaceae

**DOI:** 10.1038/s41438-019-0160-4

**Published:** 2019-06-15

**Authors:** Zhao-Yu Liu, Jian-Feng Xing, Wei Chen, Mei-Wei Luan, Rui Xie, Jing Huang, Shang-Qian Xie, Chuan-Le Xiao

**Affiliations:** 1 Hainan Key Laboratory for Biology of Tropical Ornamental Plant Germplasm, Institute of Tropical Agriculture and Forestry, Hainan University, 570228 Haikou, China; 2 State Key Laboratory of Ophthalmology, Zhongshan Ophthalmic Center, Sun Yat-sen University, 510060 Guangzhou, China; 3 Inner Mongolia Academy of Agricultural and Animal Husbandry Sciences, 010031 Huhhot, China; 4 Department of Agronomy, College of Agriculture, Purdue University, West Lafayette, IN 47907 USA

**Keywords:** DNA methylation, Plant genetics, DNA sequencing

## Abstract

Eukaryotic DNA methylation has been receiving increasing attention for its crucial epigenetic regulatory function. The recently developed single-molecule real-time (SMRT) sequencing technology provides an efficient way to detect DNA N6-methyladenine (6mA) and N4-methylcytosine (4mC) modifications at a single-nucleotide resolution. The family Rosaceae contains horticultural plants with a wide range of economic importance. However, little is currently known regarding the genome-wide distribution patterns and functions of 6mA and 4mC modifications in the Rosaceae. In this study, we present an integrated DNA 6mA and 4mC modification database for the Rosaceae (MDR, http://mdr.xieslab.org). MDR, the first repository for displaying and storing DNA 6mA and 4mC methylomes from SMRT sequencing data sets for Rosaceae, includes meta and statistical information, methylation densities, Gene Ontology enrichment analyses, and genome search and browse for methylated sites in NCBI. MDR provides important information regarding DNA 6mA and 4mC methylation and may help users better understand epigenetic modifications in the family Rosaceae.

## Introduction

DNA methylation, which is the addition of a methyl group to a DNA nucleotide, plays an important role in biological processes due to the resulting changes in DNA structure and topology^1^. Methylation on the fifth position of the cytosine pyrimidine ring (5-methylcytosine, 5mC) has been the focus of research on eukaryotic genome distribution and is an important epigenetic marker closely related to transcription^2–5^. Methylations on the sixth position of the adenine purine ring (N6-methyladenine, 6mA) and on the fourth position of the cytosine pyrimidine ring (N4-methylcytosine, 4mC) are minimal in eukaryotes and can only be detected using highly sensitive technologies. Recently, high-throughput sequencing technologies have been developed that provide highly efficient and large-scale solutions for identifying DNA methylation modifications. As a result, several researchers have observed the unexpected presence of 6mA in a large number of eukaryotic organisms, including multiple fungal species^6,7^, plants (*Arabidopsis thaliana*^1^*, Chlamydomonas reinhardtii*^8^, and *Oryza sativa*^9,10^), animals (*Drosophila melanogaster*^11^, *Mus musculus*^12^, zebrafish, and pigs^13^), and even *Homo sapiens*^14^.

The single-molecule real-time sequencing (SMRT) technology, a mainstream platform of third-generation sequencing, is prevalently applied due to the advantages of long-read sequencing and detectable DNA modification^15,16^. DNA 6mA and 4mC modifications have been detected by SMRT at a single-nucleotide resolution and single-molecule level based on variances in interpulse duration (IPD) between two successive base incorporations in modified sites of a DNA template^17^. For 6mA identification, SMRT sequencing has advantages compared with other methods^18,19^, such as liquid chromatography coupled with tandem mass spectrometry (LC-MS/MS)^20^, 6mA immunoprecipitation sequencing (6mA-IPseq)^13^, and certain restriction enzyme-based 6mA sequencing (6mA-REseq)^21^. SMRT sequencing has provided important information regarding the presence of 6mA and 4mC modifications and has greatly improved the genome-wide analysis of DNA modifications in eukaryotes.

Rosaceae is a large horticultural plant family consisting of more than 2500 species from 90 genera. Many species are economically important and produce edible fleshy fruits and nuts or serve as ornamentals^22,23^. To date, the Genome Database for Rosaceae (GDR, https://www.rosaceae.org) is the most comprehensive database of curated genomic, genetic, and breeding data for the family Rosaceae. GDR provides a valuable online resource and analysis tool for the Rosaceae community^24^. DNA methylation using SMRT has been extensively researched in recent years, and DNA methylation in the family Rosaceae has been shown to play an important role in regulating the process of fruit development and various abiotic stress responses^23,25,26^. Although there are two databases involved, DNA modification with SMRT technology has been released for the general species (MethSMRT)^27^ and chemical properties (DNAmod)^28^, respectively. An integrated database that facilitates the exploration of Rosaceae DNA methylation and provides an important complement to GDR is still lacking.

Recently, two species in the family Rosaceae (*Fragaria vesca*^29^ and *Rosa chinensis*^30^) were sequenced using SMRT technology. Thus, the published sequencing data can be analyzed for DNA 6mA and 4mC modifications. In this study, we present the DNA modification database for Rosaceae (MDR), an integrative platform for storing, analyzing, and visualizing DNA 6mA and 4mC methylation from SMRT sequencing data. MDR provides a user-friendly interface to host, browse, search, and download 6mA and 4mC profiles and offers a genome browser to visualize DNA methylation sites and display related coverage and gene annotations. The MDR database is publicly available at http://mdr.xieslab.org.

## Usage and access

**Fig. 1:**
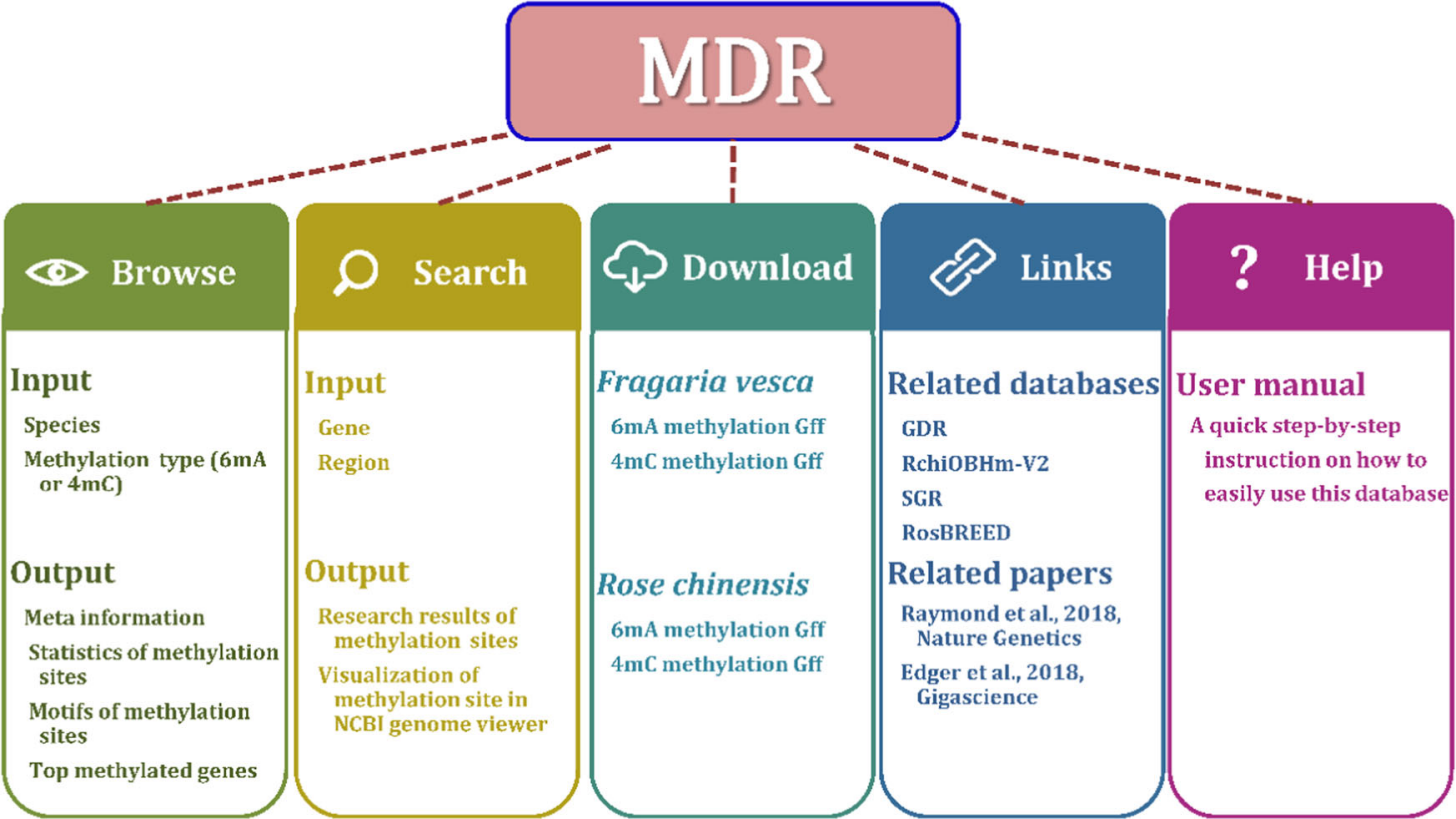
Structure and contents of MDR

MDR provides multiple interface pages, including Home, Browse, Search, Download, Help, and Links, to give information on 6mA and 4mC modifications in Rosaceae. The main structure of MDR is shown in Fig. 1.

### Browse

Meta and statistical information on *F. vesca* and *R. chinensis* can be easily found on the Browse Page. The Browse results provide: (i) meta information for SMRT sequencing data, including organism name and cultivated varieties, reference genome for alignment, SMRT sequencing instrument, BioProject accession, tissue source, and title of PubMed article (Fig. S1); (ii) 6mA and 4mC distributions and densities on chromosomes and genomic feature locations (Fig. S2); (iii) coverage of reads at 6mA or 4mC methylated sites and score for DNA methylation detection (Fig. S3); (iv) consensus sequence motif in 6mA or 4mC methylated sites (Fig. S4); (v) top methylation density genes in each species, which lists the 6mA or 4mC density of genes, 5′UTR, CDS, Intron, and 3′UTR regions (Fig. S5); and (vi) Gene Ontology (GO) enrichment analysis for genes with high methylation levels (Fig. S6).

### Search

**Fig. 2:**
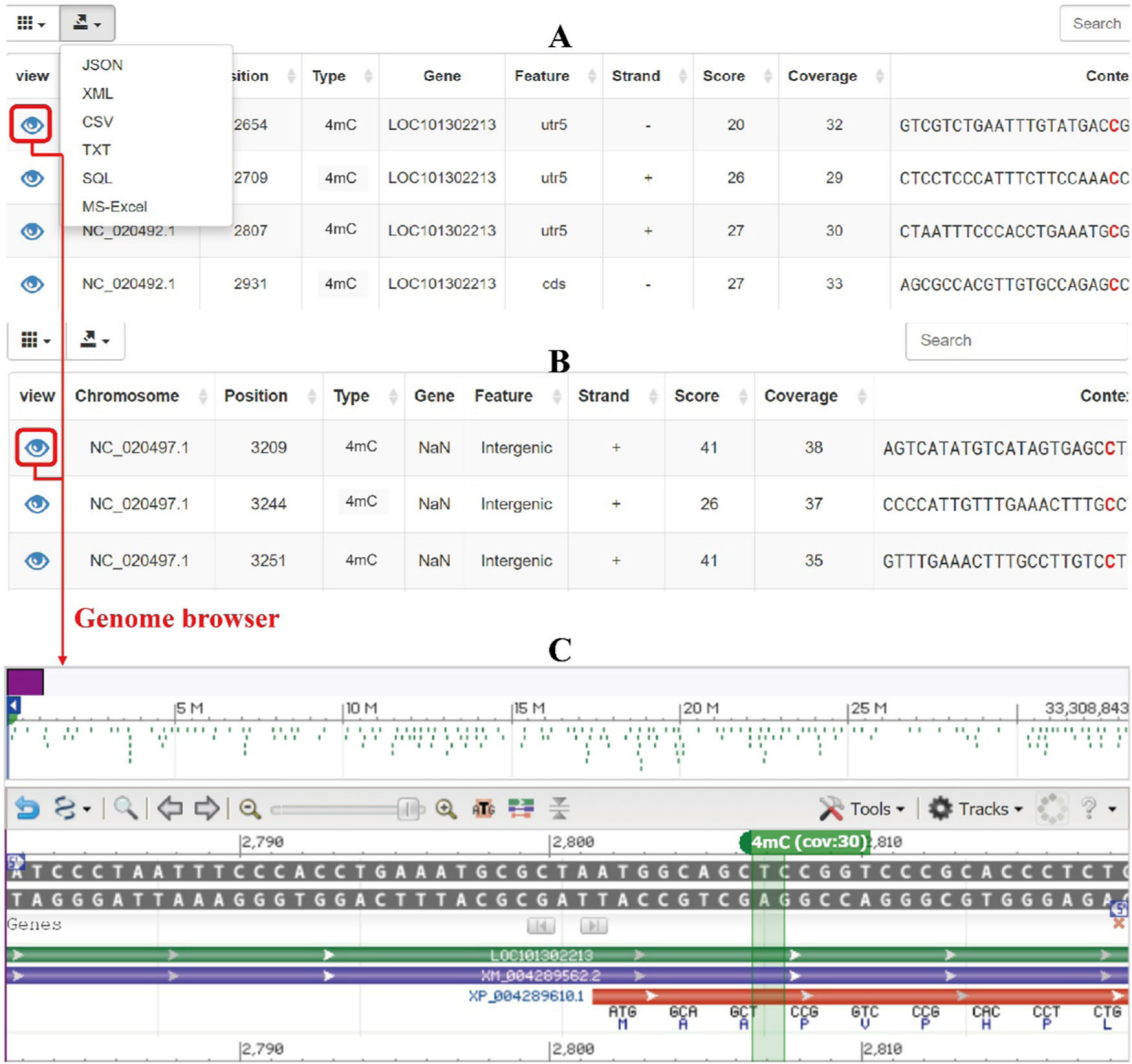
Output of query and visualization of methylation sites. (**a**) Output of query by gene, (**b**) output of query by genomic region, (**c**) visualization of methylation sites in genome browser

Two query fields are used for searching 6mA and 4mC modifications on this page. Search by gene (i): select one species and methylation type and enter one gene symbol to query. All methylated sites of the gene of interest are listed in the query results (Fig. 2a). Search by genomic region (ii): the methylated sites in the queried genomic region are shown against the selected chromosome in the query output (Fig. 2b). The outputs from the above two search patterns are displayed in a table, which includes methylated site position, methylation type, gene symbol, gene feature, strand, coverage, score, and sequence context of the methylated site (Fig. 2a, b). Users can sort the table by clicking the column names, and selected custom columns can be displayed. In addition, query results can be exported with one custom file format (Json, XML, CSV, TXT, SQL, and Excel, Fig. 2).

### Genome browser

The purpose of this browser is to visualize DNA 6mA and 4mC modification sites in the reference genome. MDR provides a genome browser in NCBI that allows users to view a custom methylated site with annotation information (Fig. 2c). The browser includes a variety of data tracks and allows users to choose tracks of interest and then zoom and scroll along any region of the genome (Fig. S7). Reference and annotated tracks are displayed at the top and bottom of the browser, respectively (Fig. S7). The location of the methylation site in the genome is marked with green, and the methylation type and coverage are displayed (Fig. 2c). This output provides an interactive and user-friendly methylome browser for each methylation site.

### Download and link

The download page provides an interface to obtain the 6mA and 4mC methylation files from SMRT sequencing datasets in addition to the annotation file used for methylation identification. MDR also lists other valuable Rosaceae databases and *F. vesca* and *R. chinensis* reference papers on the Links page.

## Discussion

MDR is the first repository for displaying and storing DNA 6mA and 4mC methylomes from SMRT sequencing data sets for Rosaceae. DNA methylation is an important type of epigenetic modification and is tightly linked to economically valuable traits in Rosaceae plants^23,25,26^. MDR provides 6mA and 4mC meta information, genomic statistical data, and detailed identifying information for each methylated site. The current version of MDR contains all publicly available SMRT data sets for Rosaceae species, including *F. vesca*^29^ and *R. chinensis*^30^. New SMRT data sets will be developed and released soon. We will continue updating MDR and will include more DNA methylomes for new Rosaceae data sets in the near future.

**Fig. 3:**
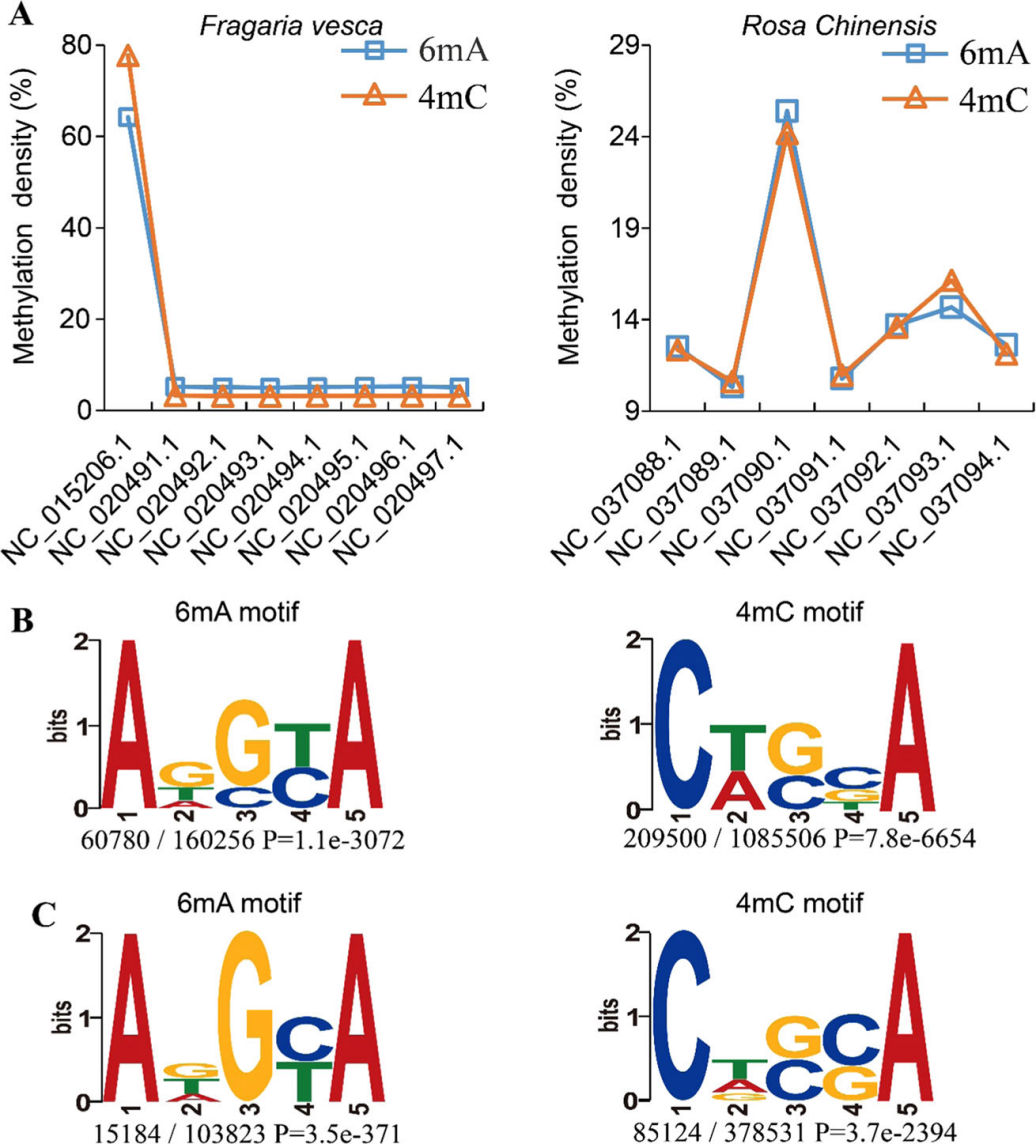
Comparison of 6mA and 4mC modifications in *F. vesca* and *R. chinensis*. **a** Percentage of methylation density at 6mA and 4mC in *F. vesca* and *R. chinensis*, (**b**) 6mA motif (ADSYA) and 4mC motif (CWSBA) in *F. vesca*, (**c**) 6mA motif (ADGYA) and 4mC motif (CDSSA) in *R. chinensis*. Sequence logos represent the consensus motifs containing methylation sites. Motif sequence numbers relative to the total number of methylation sites and the corresponding P-values (generated by MEME) are shown under the logos

MDR reveals the global patterns of 6mA and 4mC DNA methylation for Rosaceae, which may help users understand epigenetic modifications on a macroscopic scale. For example, when comparing the distributions of 6mA and 4mC in genomes and consensus motifs in the MDR database, we found multiple trends. First, 6mA and 4mC methylation densities were similar between *F. vesca* and *R. chinensis* chromosomes (Fig. 3a). The correlation coefficients were 0.99 (*p*-value < 2.2e−16) and 0.98 (*p*-value = 2.271e−05) between 6mA and 4mC in *F. vesca* and *R. chinensis*, respectively (Supplementary_Inf01). Furthermore, 6mA and 4mC modification was detected at a high degree in only one chromosome of each species, NC_015206.1 (*F. vesca*) and NC_037090.1 (*R. chinensis*) (Fig. 3a). Interestingly, we found that the chromosome NC_015206.1 in *F. vesca* had the smallest chromosome size, but its relative gene number (gene number/chr_size) was highest (Table S1). A similar result was also found for chromosome NC_037090.1 in *R. chinensis* (Table S2). To further validate this conclusion, we analyzed the DNA 6mA modification results of *A. thaliana*^10^ and *H. sapiens*^14^ against the corresponding reference genomes. The results were similar to those of *F. vesca* and *R. chinensis* (Table S3 and Table S4). The above results indicated that a high intensity of 6mA and 4mC on one chromosome might be associated with chromosome features such as chromosome size and the relative gene number (Table S1, S2, S3 and S4). In addition, conserved motif sequences were similar among the 6mA and 4mC modifications (Fig. 3b, c). The 4 bp sequence downstream of the methylated site is the base adenine (A) in 6mA and 4mC, such as the motif sequences ADSYA (6mA) and CWSBA (4mC) in *F. vesca* (Fig. 3b) and ADGYA (6mA) and CDSSA (4mC) in *R. chinensis* (Fig. 3c). Similar distribution patterns and conserved sequences suggest that DNA 6mA and 4mC may share the same generative mechanism. It has been reported that DNA methyltransferases modify both adenine residues at position N6 and cytosine residues at position N4 in prokaryotes^31,32^. Furthermore, we found that genes with high 6mA and 4mC methylation were significantly enriched in similar biological processes, cellular components, and molecular functions in *F. vesca*, such as the process of photosynthesis, chloroplast thylakoid membranes, and structural constituents of ribosome ontology (Fig. S8 and Supplementary_Inf03). This result suggests that both types of DNA methylation may serve a similar function or play roles that are complementary to each other in epigenetic regulation.

For Rosaceae, GDR is the most important resource database^24^. GDR was first released in 2003, and the amount of collected data have increased drastically in the last five years^22,24^. It has grown to provide publicly available genomic, genetic, and breeding data and multiple versions of whole genome assembly and annotation data for 14 species in the family Rosaceae. However, the genome-wide distribution patterns of DNA 6mA and 4mC modifications are still not well understood in Rosaceae. MDR contributes to the exploration of DNA methylation in Rosaceae and provides an important complement to GDR. Compared with the general DNA methylation database MethSMRT, which includes 149 species of prokaryotes and 7 model species of eukaryotes^27^, and DNAmod, which annotates the chemical properties of 6mA and 4mC^28^, MDR is a specialized and integrated database that focuses on DNA 6mA and 4mC modification profiling for Rosaceae. It provides in-depth analysis results, such as methylation density of custom genes, GO enrichment analyses, genome searches in NCBI, and comparisons between *F. vesca* and *R. chinensis*. Our goal is for MDR to become an important resource for DNA 6mA and 4mC methylation in Rosaceae.

## Materials and methods

### Data resources

Raw SMRT sequencing data h5 files for two horticultural plants, *F. vesca*^29^ and *R. chinensis*^30^, were downloaded from the NCBI Sequence Read Archive^33^. Two types of h5 files are used to detect DNA modification: (1) sequence reads, which are mapped against the reference genome to locate methylation sites; and (2) polymerase kinetics information, which is used for identifying methylated sites^17^. To date, we have collected all published SMRT sequencing datasets for Rosaceae in this database (Table 1). The reference genomes FraVesHawaii_1.0^34^ and RchiOBHm-V2^30^, obtained from NCBI, were used to align sequence reads and gene annotations for *F. vesca* and *R. chinensis*.

**Table 1:** Data resources used in the MDR database

Species	Cultivar	ProjectID	Total length of subreads (Gb)	Coverage	Reference genome
*F. vesca*	Hawaii-4	PRJNA383733	123.70	81	https://www.ncbi.nlm.nih.gov/genome/3314
*R. chinensis*	Old Blush	PRJNA413292	109.74	80	https://www.ncbi.nlm.nih.gov/genome/11715

### Data processing

**Fig. 4:**
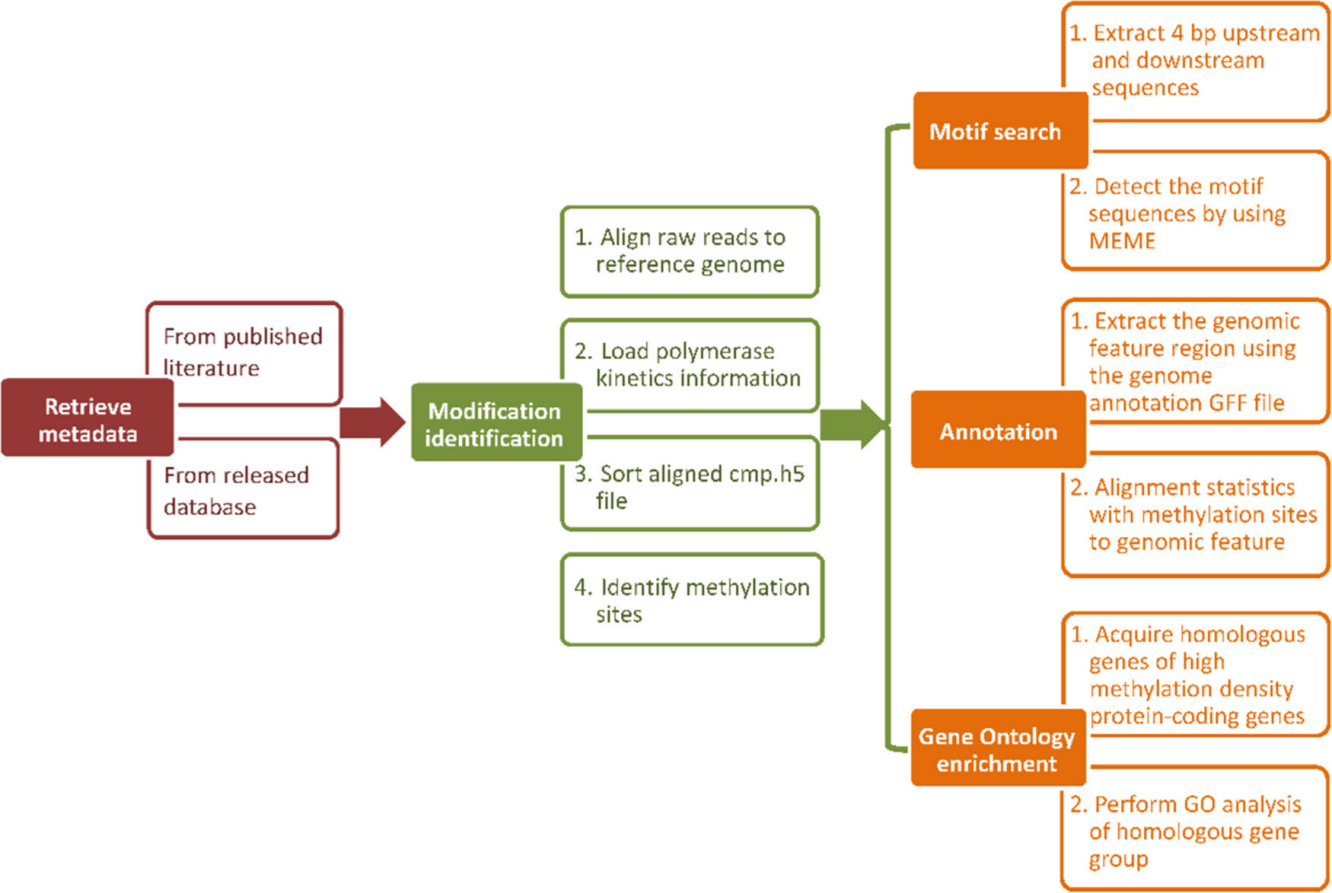
Analysis pipeline in MDR

We used the uniform analysis pipeline to process the *F. vesca* and *R. chinensis* datasets in MDR. The PacBio SMRT analysis platform (version: 2.3.0) was used to detect DNA 6mA and 4mC modifications (http://www.pacb.com/products-and-services/analyticalsoftware/smrt-analysis/analysis-applications/epigenetics/). For data analysis, raw data files in h5 format were first downloaded and filtered using filter_plsh5.py to remove sequencing adapters, short reads (defined as read lengths less than 50 nucleotides), or reads with a low quality region (read score <0.75 by default). Filtered reads were aligned to the reference genome using pbalign v0.2.0.1. Then, polymerase kinetics data were loaded by loadChemistry.py and loadPulses scripts. The aligned data sets were sorted using cmph5tools. 6mA and 4mC modifications were identified using ipdSummary.py script with ‘--methylFraction --identify 6mA, 4mC’. In summary, the analysis pipeline used in MDR is shown in Fig. 4.

### Bioinformatics analysis

We subdivided genome features into 5’UTR, CDS, Intron, 3’UTR, and Intergenic categories according to the annotation files of the reference genome. Genome features were extracted by the GenomicFeatures^35^ R package and BEDTools v2.27.1^36^. Modification sites were classified into corresponding genome features to further analyze the distribution of 6mA and 4mC sites in detail. Sequences between 4 bp upstream and 4 bp downstream of each modification site were extracted to predict conserved motifs using MEME^37^. To explore the functions of high DNA methylation genes, we analyzed the GO of the top 200 methylation density genes in each species. Due to the lack of GO terms for *F. vesca* and *R. chinensis*, we aligned the amino acid sequences of the top methylated genes with *A. thaliana* and acquired functional homologous genes using blastp^38^. Then, the homologous top methylated genes were used to perform GO enrichment analyses.

### Database implementation

The database is supported by Django (https://www.djangoproject.com/) and Apache (https://www.apache.org/). MySQL (https://www.mysql.com/) is used for data management and organization in MDR. To provide a smooth and friendly user interface, bootstrap (https://github.com/twbs/bootstrap) was used to beautify the interface, and statistical results are displayed using bootstrap-table (https://github.com/wenzhixin/bootstrap-table/issues/1765) and echarts (https://github.com/apache/incubator-echarts). NCBI Sequence Viewer (https://www.ncbi.nlm.nih.gov/tools/sviewer/) was used as the genome browser to visualize 6mA and 4mC methylation sites against the references and annotation information for *F. vesca* and *R. chinensis*.

## Supplementary information

Supplementary table and figure

Supplementary inf01

Supplementary inf02

Supplementary inf03

## Data Availability

The data sets generated and/or analyzed during the current study are available in the MDR database, http://mdr.xieslab.org.
