# Supplementary material for: MDR: an integrative DNA N6-methyladenine and N4-methylcytosine modification database for Rosaceae
Source: Hortic Res. 2019 Jun 15;6:78. doi: 10.1038/s41438-019-0160-4 (PMC6572862; doi:10.1038/s41438-019-0160-4)
Supplement: Supplementary file 1 — Supplementary table and figure [file 41438_2019_160_MOESM1_ESM.docx]

Table S1. Chromosomes statistics in *F. vesca*

| Chromosome | 6mA density (%) | 4mC density (%) | chr_size (Mb) | Gene  number | Gene_number  /chr_size |  |
| --- | --- | --- | --- | --- | --- | --- |
| **NC_015206.1** | **0.6421** | **0.7770** | **0.16** | **130** | **812.50** | |
| NC_020491.1 | 0.0517 | 0.0326 | 22.68 | 2869 | 126.50 | |
| NC_020492.1 | 0.0507 | 0.0313 | 33.31 | 4115 | 123.54 | |
| NC_020493.1 | 0.0499 | 0.0313 | 27.88 | 3689 | 132.32 | |
| NC_020494.1 | 0.0511 | 0.0317 | 23.29 | 2672 | 114.73 | |
| NC_020495.1 | 0.0517 | 0.0317 | 29.33 | 3739 | 127.48 | |
| NC_020496.1 | 0.0522 | 0.0321 | 38.22 | 5036 | 131.76 | |
| NC_020497.1 | 0.0505 | 0.0322 | 23.4 | 3229 | 137.99 | |

Table S2. Chromosomes statistics in *R. chinensis*

| Chromosome | 6mA density  (%) | 4mC density  (%) | chr_size  (Mb) | Gene  number | Gene_number  /chr_size |
| --- | --- | --- | --- | --- | --- |
| NC_037088.1 | 0.1251 | 0.1236 | 68.49 | 5116 | 74.70 |
| NC_037089.1 | 0.1032 | 0.1062 | 88.56 | 6717 | 75.85 |
| **NC_037090.1** | **0.2539** | **0.2418** | **49.74** | **3835** | **77.10** |
| NC_037091.1 | 0.1078 | 0.1097 | 67.24 | 4731 | 70.36 |
| NC_037092.1 | 0.1370 | 0.1360 | 89.95 | 6540 | 72.71 |
| NC_037093.1 | 0.1467 | 0.1615 | 69.64 | 5312 | 76.28 |
| NC_037094.1 | 0.1262 | 0.1212 | 70.29 | 5107 | 72.66 |

Table S3. Chromosomes statistics in *A. thaliana*

| Chromosome | 6mA density  (%) | chr_size  (Mb) | Gene  number | Gene number  /chr size |
| --- | --- | --- | --- | --- |
| NC_003070.9 | 0.0306 | 30.43 | 8,771 | 288.24 |
| NC_003071.7 | 0.0513 | 19.7 | 5,265 | 267.26 |
| NC_003074.8 | 0.0398 | 23.46 | 6,544 | 278.94 |
| NC_003075.7 | 0.0415 | 18.59 | 5,007 | 269.34 |
| NC_003076.8 | 0.0361 | 26.98 | 7,468 | 276.80 |
| NC_037304.1(MT) | 0.0783 | 0.37 | 276 | 745.95 |
| **NC_000932.1(Pltd)** | **0.1433** | **0.15** | **129** | **860.00** |

Table S4. Chromosomes statistics in *H. sapiens*

| Chromosome | 6mA density  (%) | Chr_size  (Mb) | Gene  number | Gene number  /chr size |
| --- | --- | --- | --- | --- |
| NC_000001.11 | 0.0527 | 248.96 | 3723 | 14.95 |
| NC_000002.12 | 0.0531 | 242.19 | 2690 | 11.11 |
| NC_000003.12 | 0.0513 | 198.3 | 2090 | 10.54 |
| NC_000004.12 | 0.0510 | 190.22 | 1638 | 8.61 |
| NC_000005.10 | 0.0508 | 181.54 | 1814 | 9.99 |
| NC_000006.12 | 0.0524 | 170.81 | 2123 | 12.43 |
| NC_000007.14 | 0.0515 | 159.35 | 1881 | 11.80 |
| NC_000008.11 | 0.0521 | 145.14 | 1494 | 10.29 |
| NC_000009.12 | 0.0530 | 138.4 | 1564 | 11.30 |
| NC_000010.11 | 0.0536 | 133.8 | 1539 | 11.50 |
| NC_000011.10 | 0.0505 | 135.09 | 2095 | 15.51 |
| NC_000012.12 | 0.0526 | 133.28 | 1835 | 13.77 |
| NC_000013.11 | 0.0576 | 114.36 | 910 | 7.96 |
| NC_000014.9 | 0.0512 | 107.04 | 1480 | 13.83 |
| NC_000015.10 | 0.0530 | 101.99 | 1270 | 12.45 |
| NC_000016.10 | 0.0559 | 90.34 | 1469 | 16.26 |
| NC_000017.11 | 0.0529 | 83.26 | 1894 | 22.75 |
| NC_000018.10 | 0.0504 | 80.37 | 689 | 8.57 |
| NC_000019.10 | 0.0511 | 58.62 | 1976 | 33.71 |
| NC_000020.11 | 0.0532 | 64.44 | 1020 | 15.83 |
| NC_000021.9 | 0.0635 | 46.71 | 570 | 12.20 |
| NC_000022.11 | 0.0554 | 50.82 | 835 | 16.43 |
| NC_000023.11(X) | 0.0234 | 156.04 | 1311 | 8.40 |
| NC_000024.10(Y) | 0.0240 | 57.23 | 188 | 3.28 |
| **NC_012920.1(MT)** | **0.1844** | **0.016** | **37** | **2312** |


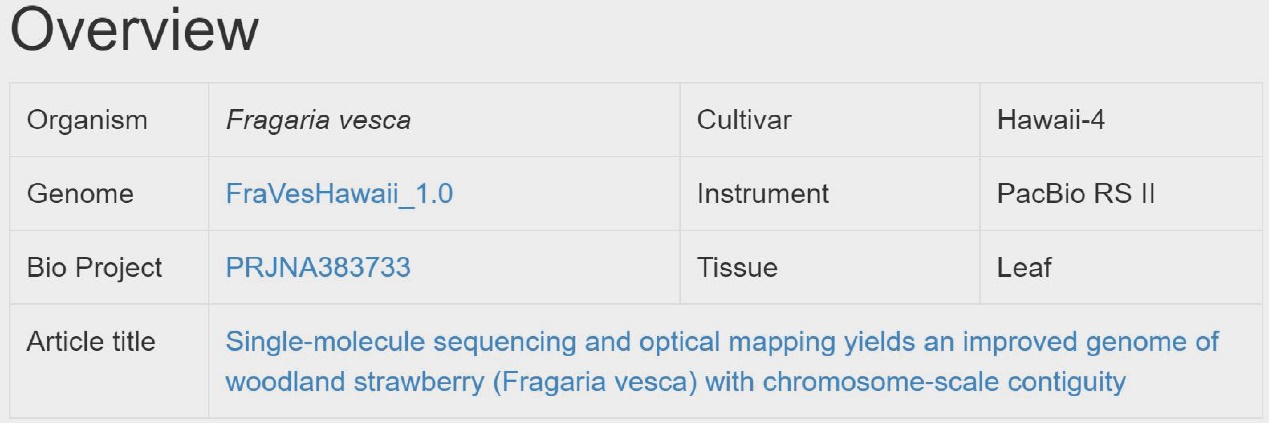


Figure S1. Meta information of SMRT sequencing data


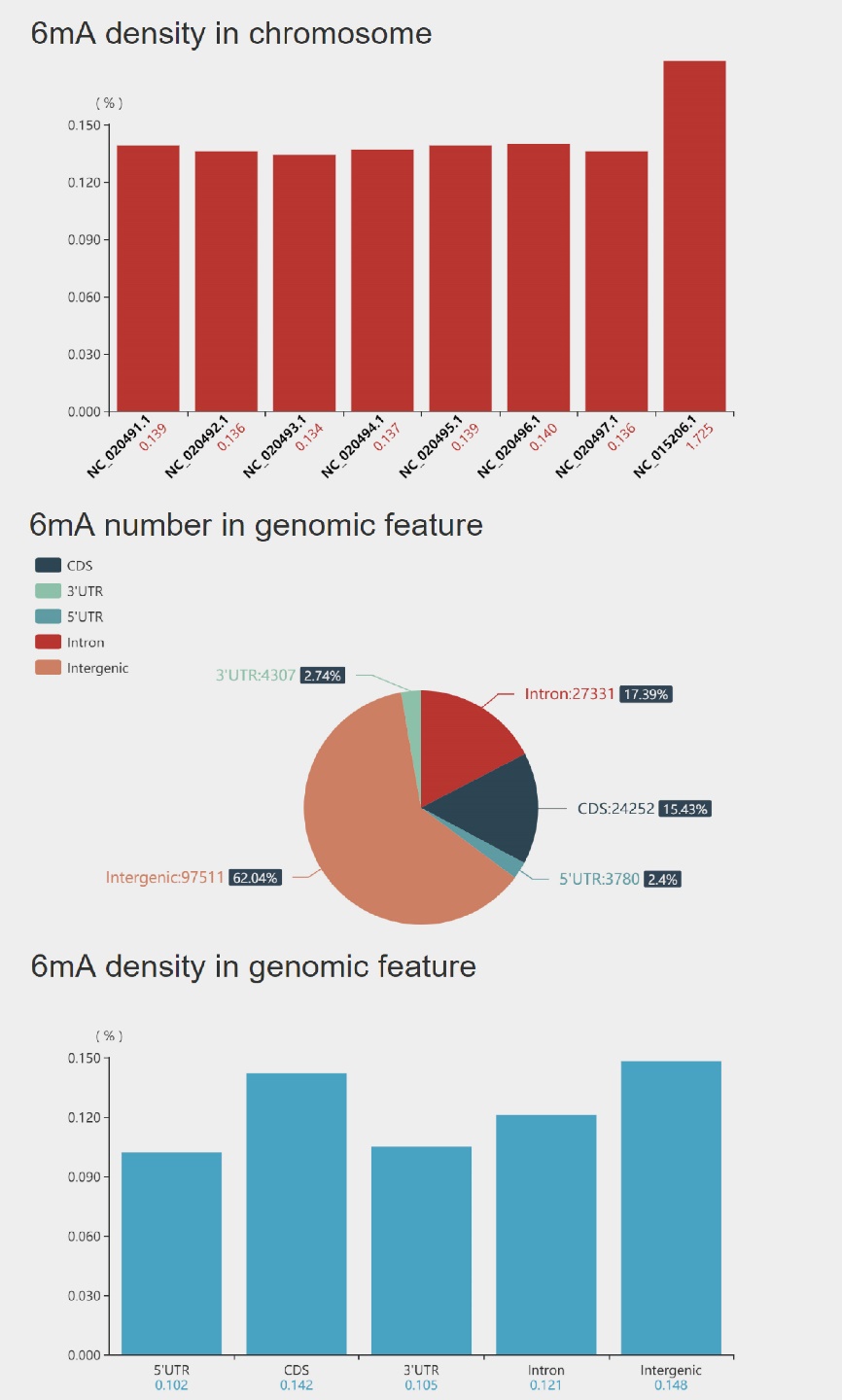


Figure S2. Statistical information of methylation sites


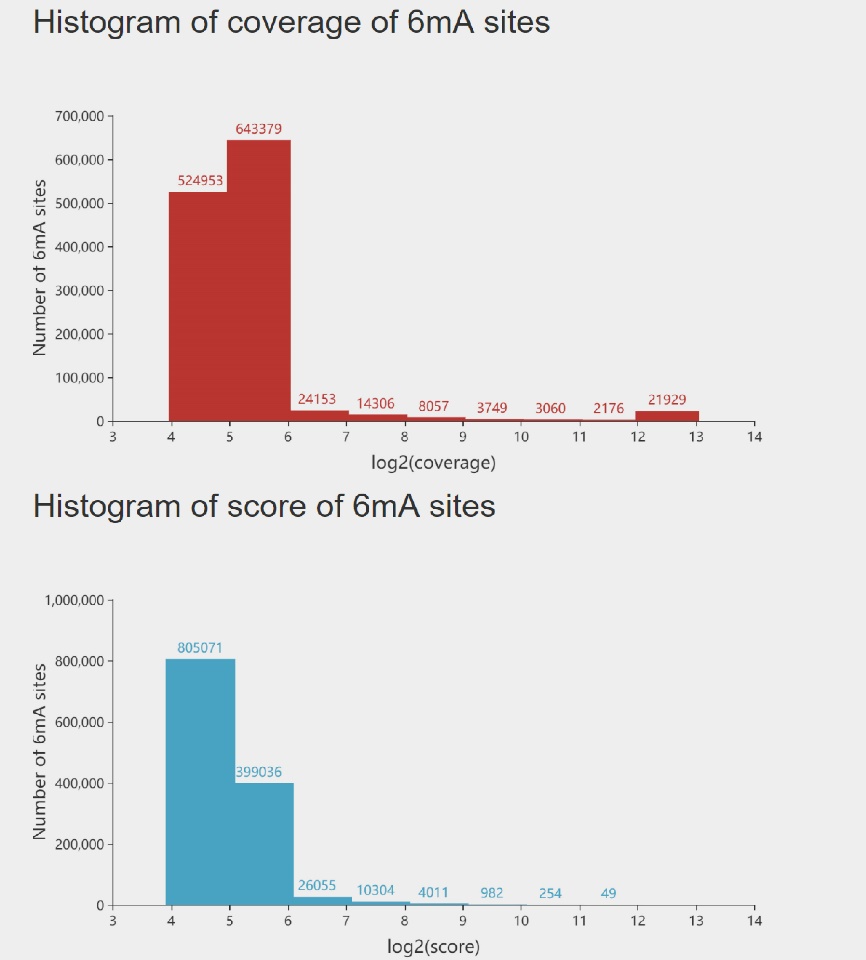


Figure S3. Coverage and score of methylation sites


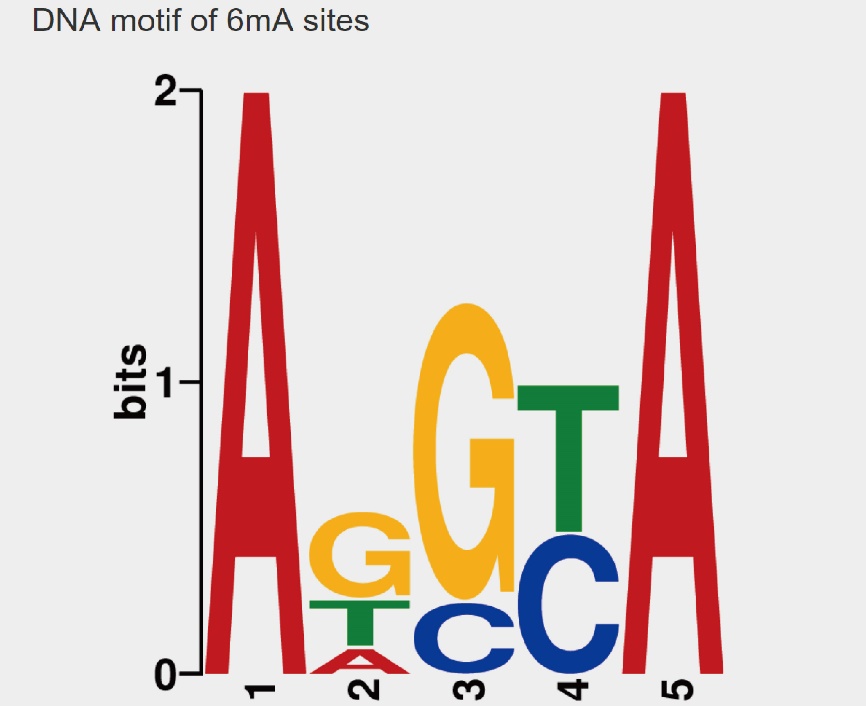


Figure S4. Consensus sequence motif of methylation sites


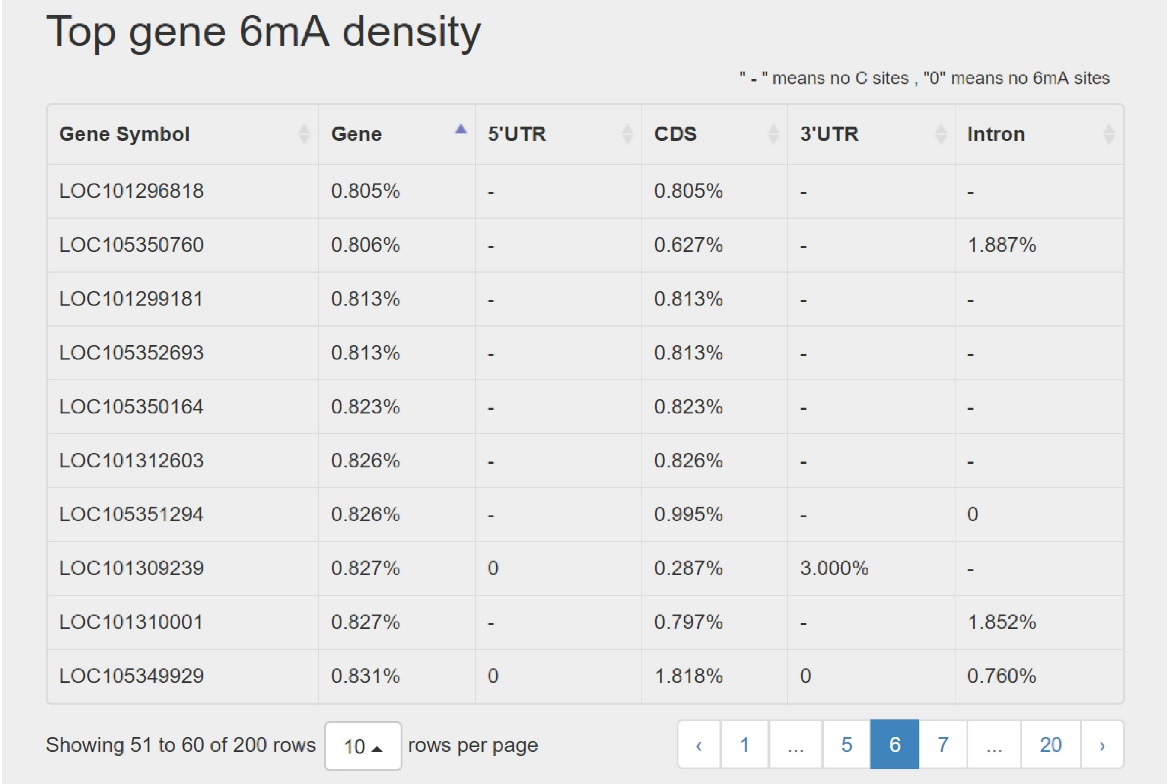


Figure S5. Methylation density of genomic features in top methylated genes


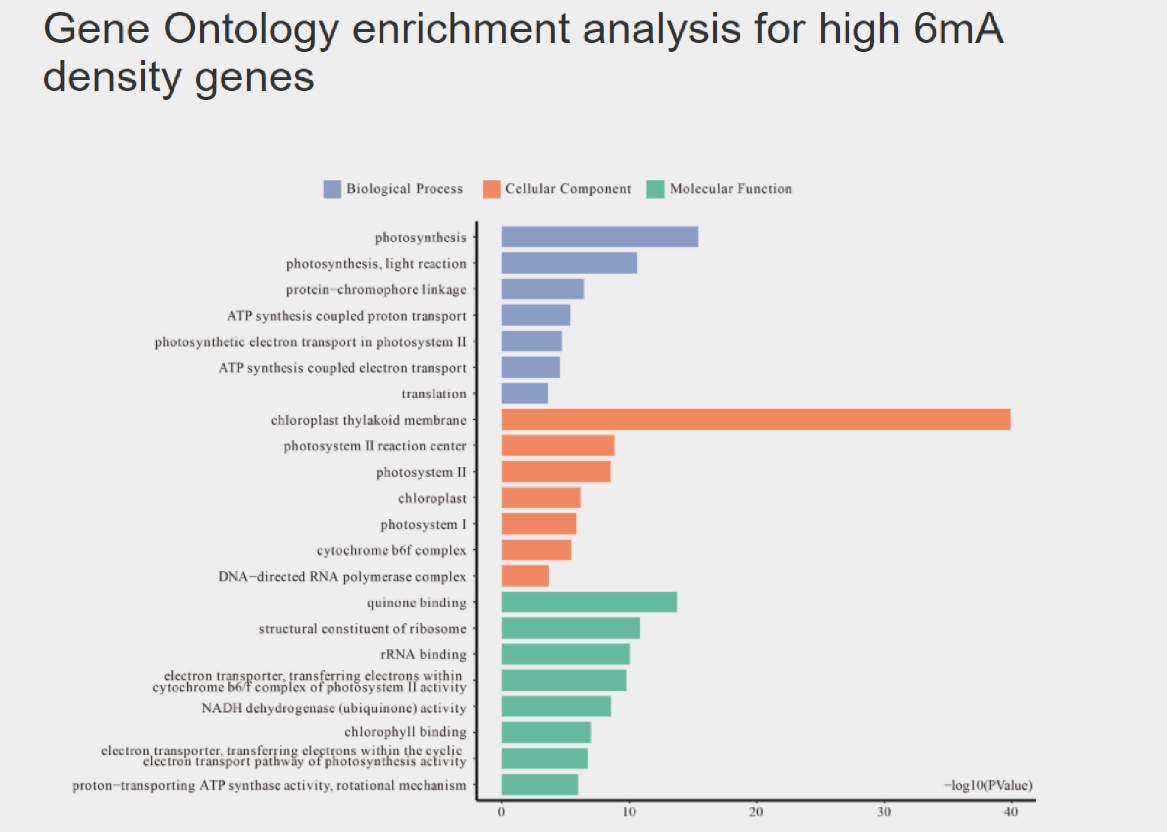


Figure S6. Gene Ontology enrichment analysis for high methylation level genes


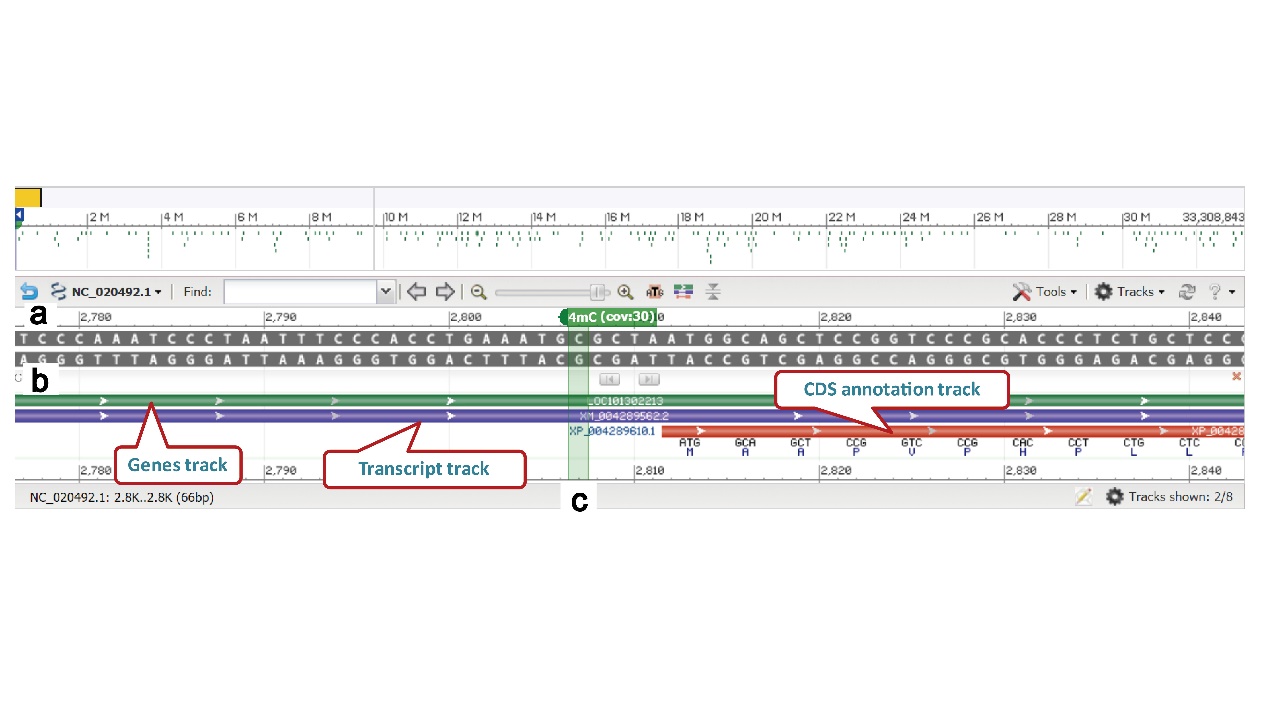
Figure S7. Genome browser (a: Reference genome track with forward and reverse strand in two gray bands; b: Genome annotation track including genes track in green band, transcript track in purple band, CDS track in red band; c: methylation site with green)


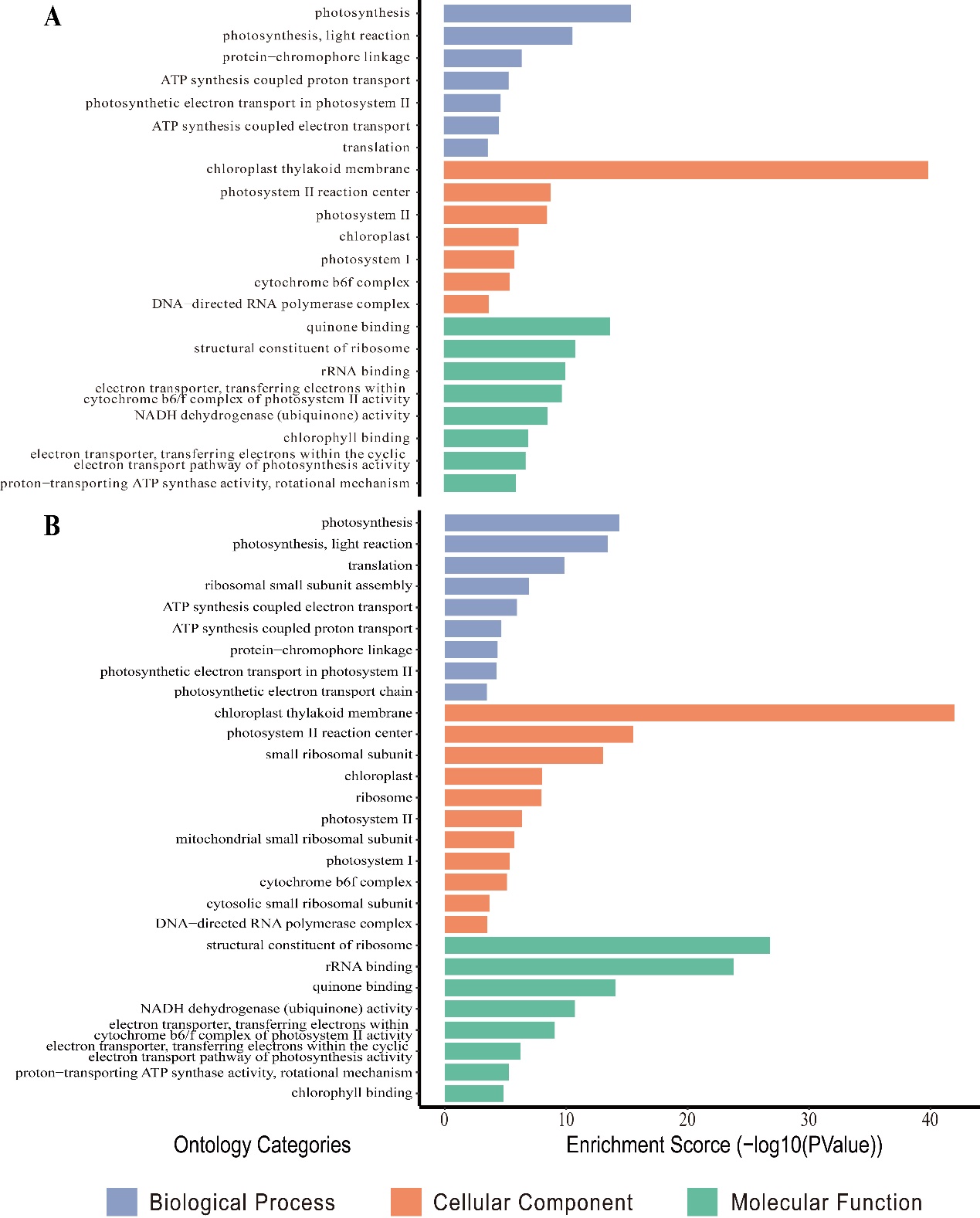


Figure S8. GO enrichment category of TOP 200 genes with higher methylation density in *Fragaria vesca* (A: genes with higher 6mA modification density; B: genes with higher 4mC modification density).
